# Extraction of heavy metals from copper tailings by ryegrass (*Lolium perenne* L.) with the assistance of degradable chelating agents

**DOI:** 10.1038/s41598-024-58486-w

**Published:** 2024-04-01

**Authors:** Weiwei Wang, Jinchun Xue, Liping Zhang, Min He, Jiajia You

**Affiliations:** 1 School of Chemical and Environmental Engineering, China University of Mining and Technology-Beijing, Beijing, 100083 China; 2 School of Energy and Mechanical Engineering, Jiangxi University of Science and Technology, Nanchang, 330013 Jiangxi China; 3 School of Software Engineering, Jiangxi University of Science and Technology, Nanchang, 330013 China

**Keywords:** Copper tailings, Heavy metal, Phytoremediation, Biodegradable chelating agents, Ryegrass, Ecology, Environmental sciences

## Abstract

Heavy metal contamination is an urgent ecological governance problem in mining areas. In order to seek for a green and environmentally friendly reagent with better plant restoration effect to solve the problem of low efficiency in plant restoration in heavy metal pollution soil. In this study, we evaluated the effects of three biodegradable chelating agents, namely citric acid (CA), fulvic acid (FA) and polyaspartic acid (PASP), on the physicochemical properties of copper tailings, growth of ryegrass (*Lolium perenne* L.) and heavy metal accumulation therein. The results showed that the chelating agent application improved the physicochemical properties of copper tailings, increased the biomass of ryegrass and enriched more Cu and Cd in copper tailings. In the control group, the main existing forms of Cu and Cd were oxidizable state, followed by residual, weak acid soluble and reducible states. After the CA, FA or PASP application, Cu and Cd were converted from the residual and oxidizable states to the reducible and weak acid soluble states, whose bioavailability in copper tailings were thus enhanced. Besides, the chelating agent incorporation improved the Cu and Cd extraction efficiencies of ryegrass from copper tailings, as manifested by increased root and stem contents of Cu and Cd by 30.29–103.42%, 11.43–74.29%, 2.98–110.98% and 11.11–111.11%, respectively, in comparison with the control group. In the presence of multiple heavy metals, CA, FA or PASP showed selectivity regarding the ryegrass extraction of heavy metals from copper tailings. PCA analysis revealed that the CA-4 and PASP-7 treatment had great remediation potentials against Cu and Cd in copper tailings, respectively, as manifested by increases in Cu and Cd contents in ryegrass by 90.98% and 74.29% compared to the CK group.

## Introduction

During the mining and beneficiation of metal mines, substantial waste tailings are produced. Metal tailing brings pollution and harm to the surrounding environment and human beings, since they contain massive high concentration heavy metals^1^. Just low intake of heavy metals can produce obvious toxic side effects on the human body^2^. For example, cadmium (Cd) can accumulate in the liver and kidney tissues, causing renal absorption insufficiency^3^. Moreover, heavy metals easily accumulate in organisms via the food chain and are hardly eliminated through biodegradation, which thus threaten the human health tremendously^4^.

Phytoremediation technology for heavy metal-contaminated soil is an emerging technique aimed at extracting or deactivating heavy metals, and it has gained attention due to its environmental friendliness and relatively low cost^5,6^. However, the low bioavailability of most heavy metals in soil hinders plant extraction^7^. In the process of plant extraction, hyperaccumulator plants and tolerant plants have received widespread attention. Although hyperaccumulator plants demonstrate good performance in heavy metal extraction and transfer, their low biomass and poor environmental adaptability often limit their application^8^. In contrast, although tolerant plants have lower unit efficiency in absorbing heavy metals, their higher biomass allows them to absorb a similar amount of heavy metals as hyperaccumulator plants^9^. Therefore, to improve the absorption efficiency of tolerant plants for heavy metals, chelating agents are introduced to enhance plant extraction technology as a potential means of improving plant extraction efficiency^10^. By adding chelating agents to promote the activation of heavy metals on the soil particle surface and form more easily extractable soluble metal complexes, plant extraction can be enhanced^11^.

Chelating agents can activate heavy metal in soil and enhance plant extraction ability^12^. Choosing the appropriate chelating agent is the key to improving the efficiency of remedying heavy metal-polluted soil. Ethylenediamine tetraacetic acid (EDTA) and diethylenetriaminepentaacetic acid (DTPA) are widely used due to their strong chelation ability for heavy metals such as Cu and Cd^13^. However, they cannot be degraded in soil, causing secondary pollution and potential risks to soil and groundwater systems through irrigation or precipitation^14^. Therefore, it is crucial to screen environmentally-friendly and effective chelating agents for Cu and Cd polluted soil remediation. Our review of relevant literature found that polyaspartic acid (PASP), citric acid (CA) and fulvic acid (FA) are three chelating agents that incur little damage to plants, have strong heavy metal extractability from soil and produce eco-friendly degradation products^15–17^. As a water-soluble amino acid polymer with good biodegradability, PASP can stay dispersed and stable in solution, since its degradation products are non-toxic, harmless and can form ligands with numerous heavy metal ions^18^. CA, as a high-efficiency heavy metal chelating agent, can effectively change the bioavailability and form of heavy metals in soil through the complexation and the input of H^+^ into the soil^19^. In addition to having a high solubility for target contaminants, it also exhibits a good environmental safety. On the other hand, the application of CA can not only regulate soil pH, increase the microbial quantity and soil enzyme activity, but also activate soil nutrients to promote the nutrient absorption and growth of plants ^20^. According to a report by Fu et al.^21^ PASP and CA can effectively dissolve the complexed metals in soil and improve the bioavailability of metals, thereby facilitating the heavy metal absorption by *Echinochloa cruso* galli. FA, as one major component of soil organic matter (SOM), is easily soluble in water and has a small molecular weight, a low carbon/hydrogen ratio and a high degree of carboxylic acid^22^. It can interact with heavy metals and other pollutants in soil by ways of adsorption, covalent binding, coordination and hydrogen bonding, which vitally influences the environmental behaviors of heavy metals such as migration, morphological change, stability and bioavailability^23^. Li et al.^24^ investigated the hyperaccumulation mechanism of FA for heavy metal activation or passivation and plant response, finding that low-molecular-weight FA could promote the Cd accumulation in plants by providing more metal adsorption sites.

However, most research focuses on the effects of chelating agent types and concentrations on plant remediation of heavy metal-polluted soil, with little attention paid to the effects of chelating agent types and application concentrations on the remediation of heavy metals in copper tailings. Ryegrass (*Lolium perenne* L.), with features like wide adaptability, fast growth and easy harvesting, and enrichment of metal ions in the soil, was an important forage and green plant very suitable for remedying heavy metals^25,26^. When metal ions enter the roots of the plant, they form complexes by chelating with small organic acids secreted by the roots, which were then immobilized in the cell wall, cytoplasm, or vacuole, thereby losing their toxicity^27,28^. On the other hand, storing metal ions in root vacuoles can reduce their toxicity, prevent the transport of metal ions to the stems of the plant, enrich them on the surface of the plant roots, and prevent the diffusion and migration of metal ions in the soil^29,30^.

Previous studies have reported its use as a restorative plant for copper tailings^31^. In order to explore the effects of biodegradable chelating agents on plant extraction of Cu and Cd in copper tailings, this study takes the copper tailings from the 4# tailings pond of Dexing Copper Mine in Jiangxi Province as the research object, and through pot experiments, investigates the effects of different concentrations of chelating agents PASP, CA, or FA on the remediation efficiency of ryegrass plants, revealing their potential mechanisms on plant remediation efficiency, and then selecting the most ideal chelating agent and application ratio.

## Materials and methods

### Chelating agents, plant and copper tailings

PASP, CA and FA were selected as experimental materials, which were procured from Hengxing Chemical Reagent in Tianjin. Both CA and FA were analytically pure, while PASP was chemically pure. PASP, as a novel green treatment agent with phosphorus free, non-toxic, pollution-free and fully biodegradable properties, has a strong ion chelating ability, which can be used as a nutrient absorption enhancer in agriculture to promote crop production^32^. CA is a novel green degradable chelating agent, which is characterized by wide pH range, strong chelating ability and no ecotoxicity^33^. Meanwhile, FA is easily soluble in water and has a small molecular weight, which can adequately activate heavy metals in soil^27^.

The fresh samples of seeds of ryegrass used in the study were obtained commercially from Muyangdou Seed Research Industry Co., LTD. The initial germination percentage of the seeds was over 95%. The plant was identified and authenticated by Professor Jiaqing He in the Herbarium, Biology Department, Nanjing University, with voucher number 19103042. The collection, preservation and use of plant materials involved in this study complied with relevant institutional, national, and international guidelines and legislation.

The experimental copper tailings were collected from the 4# tailing pond (29° 02′ 46.89″ N 117° 47′ 35.98″ E) of Dexing Copper Mine, Dexing, Jiangxi, China. The cultivation soil samples were collected by five-point sampling. After removing the stone rhizome impurities and mixing, the soil samples were packed into sealed bags by quartering method, and the mass of copper tailings in the sealed bags was controlled around 60 g. Table 1 details the basic physicochemical properties of the copper tailings.

**Table 1:** Basic characteristics of copper tailings in this study.

Categories	Units	Mean ± standard deviation (n = 3)
pH (soil:water = 1:2.5)	(–)	9.01 ± 0.02
Electrical conductivity	µS cm^-1^	112.47 ± 17.34
Water content	%	4.51 ± 0.38
Cation exchange capacity	cmol kg^-1^	3.01 ± 0.19
Soil organic matter	g kg^-1^	9.62 ± 0.24
Total nitrogen	g kg^-1^	0.08 ± 0.01
Total phosphorus	g kg^-1^	0.87 ± 0.03
Total potassium	g kg^-1^	16.11 ± 1.54
Available nitrogen	mg kg^-1^	20.00 ± 10.00
Available potassium	mg kg^-1^	78.13 ± 16.78
Available phosphorus	mg kg^-1^	4.06 ± 0.57
Copper (Cu)	mg kg^-1^	837.56 ± 98.76
Cadmium (Cd)	mg kg^-1^	0.33 ± 0.02
Lead (Pb)	mg kg^-1^	23.95 ± 3.80
Zinc (Zn)	mg kg^-1^	81.80 ± 12.34

### Pot experiment

Based on the analysis of physicochemical structure characteristics of copper tailings, combined with the basic nutrient requirements for plant growth, 4% mass fraction of earthworm casts was added to each pot containing 1500 g copper tailings, in order to meet the basic conditions for plant growth. Irrigation and stabilization were sustained for one week to release fertility. The pots used in the experiment each had an upper edge diameter of 20 cm, a basal diameter of 15 cm and a height of 15 cm. To ensure soil moisture and sufficient air for plant roots, each pot was designed with a small 1-cm-diameter hole at the bottom, which was supported by the tray. On July 13 of 2022, an outdoor pot experiment was carried out on the 6th-floor rooftop of the experimental building on Nanchang Campus of Jiangxi University of Science and Technology. In each pot, 50 seeds of ryegrass were sown and, 10 days later, the seedlings were thinned to retain 30 seedlings per pot. On August 13 and September 13 of 2022, preprepared chelating agents (each 200 mL) were applied separately to the pots, while the control (CK) group was added with an equivalent volume of deionized water. The concentrations of CA and FA were 1, 4, 7 and 10 mmol L^-1^, respectively, whereas the concentrations of PASP were 1, 4, 7 and 10 g L^-1^. There were totally 13 treatments, which were denoted as CK, CA-1, CA-4, CA-7, CA-10, FA-1, FA-4, FA-7, FA-10, PASP-1, PASP-4, PASP-7 and PASP-10, respectively, and each treatment was triplicated. On November 13 of 2022, plants were harvested and copper tailings were collected, which were packed into sealed sample bags. The mass of copper tailings in each bag was controlled around 60 g.

### Sampling and sample analyses

#### Soil sample analysis

After sun-curing and drying, the soil samples were filtered through 100-mesh sieve, weighed (10 g) into a 50-mL conical flask, added with 25 mL of deionized water, shaken for 2 h to allow thorough mixing, and then let stand still. The soil pH and electrical conductivity (EC) were measured with a rapid multi-parameter tester (COMBI 5000, Germany)^34^. The content of soil organic matter (SOM) was determined by the potassium dichromate external heating method^35^. The redox potential (Eh) was assessed with FJA-6 ORP depolarization automatic analyzer^36^, while the cation exchange capacity (CEC) of soil was estimated by hexamine cobalt trichloride leaching-spectrophotometry^37^. The available Cu and Cd contents in soil were determined using DTPA-CaCl_2_-TEA system and atomic absorption spectrometry^38^. Following the BCR sequential extraction scheme, the soil Cu and Cd forms were classified into the weak acid soluble, reducible, oxidizable and residual states^39^. The contents of various forms of Cu and Cd were all quantified with an ICP-OES spectrometer (Optima 7300 DV, USA) ^40^. The soil samples were digested with HCl-HNO_3_-HF-HClO_4_ system to determine the total soil Cu and Cd contents by atomic absorption spectrometry (GB/T 17,138-1997) ^41^.

#### Plant sample analysis

The harvested ryegrass stems and leaves (aboveground part) were treated independently from the roots (underground part). Initially, the stems, leaves and roots were cleaned with distilled water and dried naturally. Then, they were placed into a 105 °C oven for 30 min to deactivate enzymes, and subsequently dried to constant weight at 75 °C. After measuring the dry weights (biomasses) of ryegrass roots, stems and leaves separately on analytical balance, the plants were artificially ground and screened through 0.15-mm sieve for later use.

The Cu and Cd contents in ryegrass roots and stems were analyzed by atomic absorption spectrophotometry^42^. The plant powder was weighed (1.00 g) into the crucible, and then placed in the Muffle furnace for 6 h of incineration at 500 °C, and then removed after cooling to ensure complete ashing of plants. The ashed plants were digested with 10 mL of HNO_3_:HCLO_4_ = 9:4 (v/v) for 24 h, and subsequently the Cu and Cd contents in leach solution were quantified with a flame atomic absorption spectrophotometer, thereby obtaining the Cu and Cd contents in the plants.

### Data analysis

The experimental data were simply processed with Excel 2010, and subjected to one-way ANOVA via SPSS 21.0 and to significant difference analysis by LSD method (*P* < 0.05). The results were plotted using Origin 9.0. The computational formulas for the accumulation (AC), total accumulation (TAC), bioconcentration factor (BCF) and transport factor (TF) of Cu and Cd in various plant parts were as follows^43,44^:

1 $${\text{AC}}_{{{\text{stem}}}} = {\text{B}}_{{\text{S}}} \times {\text{C}}_{{\text{S}}}$$

2 $${\text{AC}}_{{{\text{root}}}} = {\text{B}}_{{\text{R}}} \times {\text{C}}_{{\text{R}}}$$

3 $${\text{TAC}} = {\text{B}}_{{\text{R}}} \times {\text{C}}_{{\text{R}}} + {\text{B}}_{{\text{S}}} \times {\text{C}}_{{\text{S}}}$$

4 $${\text{BCF}}_{{{\text{stem}}}} = {\text{C}}_{{\text{S}}} /{\text{C}}_{{{\text{soil}}}}$$

5 $${\text{BCF}}_{{{\text{root}}}} = {\text{C}}_{{\text{R}}} /{\text{C}}_{{{\text{soil}}}}$$

6 $${\text{TF}} = {\text{C}}_{{\text{S}}} /{\text{C}}_{{\text{R}}}$$

where BS and BR respectively denote the biomasses of ryegrass stems and roots; CR and CS respectively denote the heavy metal (Cu, Cd) contents in roots and stems; Csoil represents the content of heavy metals (Cu, Cd) in copper tailings.

## Results

### Ryegrass growth and soil properties

**Figure 1:**
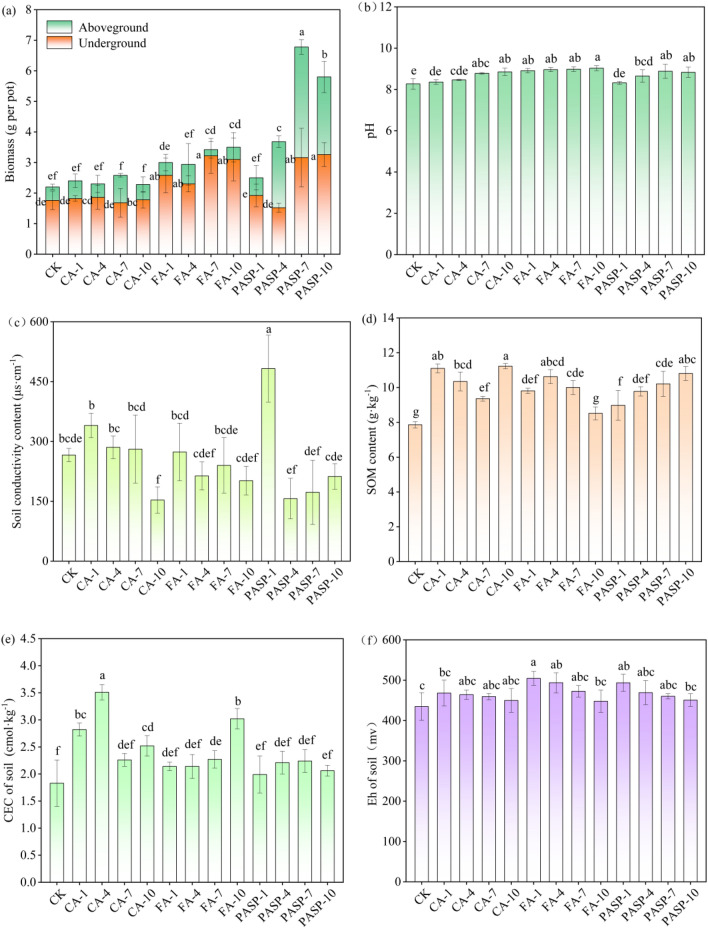
The biomass of ryegrass (**a**) and pH (**b**), soil conductivity (**c**), SOM (**d**), CEC (**e**), Eh (**f**) of copper tailings under different treatments. SOM: soil organic matter, CEC: cation exchange capacity, Eh: redox potential. Values are means ± SD (n = 3). Different letters indicate significant differences at *p* ≤ 0.05 according to the Tukey’s HSD test; values are in the order a > b > c.

Compared to the CK group, treatment with various chelating agents did not produce obvious signs of toxicity, such as leaf yellowing or dysplasia, to the ryegrass growth. As shown in Fig. 1a, the biomass in the CK group was 2.20 g/pot for the aboveground part, whereas was 1.76 g/pot for the underground part, revealing a total biomass of 3.96 g/pot. The application of chelating agents significantly enhanced the biomass of ryegrass such an enhancement effect was most pronounced with PASP at a spraying concentration of 7 g/L, where the biomasses of aboveground and underground parts increased by 208.28% and 79.55%, respectively, compared to the CK group.

After phytoremediation, the physicochemical properties of copper tailings were assessed, including pH, EC, SOM, CEC and Eh, as displayed in Fig. 1b–f. The copper tailings in the CK group had a pH of 8.27, while after application of different concentrations of chelating agents, the tailings pH values were all higher than the CK group. Nevertheless, the chelating agents’ activation effects on Cu and Cd in copper tailings were unaffected. Addition of high concentration CA significantly elevated the tailings pH from 8.27 to 8.85. Addition of FA also elevated the tailings pH, albeit the insignificant inter-concentration differences. With the increase of PASP concentration, the pH of copper tailings rose initially and then dropped. With the increase of CA concentration, the EC of copper tailings tended to increase first and then decrease. Under CA-10 treatment, the copper tailings EC was 153.17 µS/cm, which significantly decreased by 42.42% compared to the CK group. No significant differences were noted among different concentrations of CA groups. Except for the low concentration PASP, which significantly increased EC of copper tailings up to 482.67 µS/cm, the remaining treatments all exhibited EC values lower than the CK group. The SOM content of CK group was 7.86 g/kg, while after treatment with CA, FA and PASP, the SOM content of copper tailings significantly increased by up to 42.88%, 35.24% and 37.53%, respectively. Moreover, the SOM content under CA-10 treatment was 5.64% and 3.89% higher than that under FA-4 and PASP-10 treatments, respectively. Addition of chelating agents all increased the soil CEC to varying degrees, with CA-4, FA-10 and PASP-7 exhibiting the most prominent effects on enhancing the CEC of copper tailings, showing increases by 91.80%, 65.03% and 22.40%, respectively, in comparison to the CK group. Although the addition of chelating agents to copper tailings was all effective in enhancing the tailings Eh, the extent of enhancement decreased with the heightening of chelating agent concentration. FA produced a greater enhancement effect on the copper tailings Eh than PASP and CA, showing Eh increases by 16.08%, 13.57%, 8.70% and 3.04%, respectively, compared to the CK group. This indicates that the addition of CA, FA and PASP could all improve the soil reduction status and increase the soil nutrients, thereby enhancing the Eh of soil.

### Heavy metal content in soil and changes in heavy metal availability

**Figure 2:**
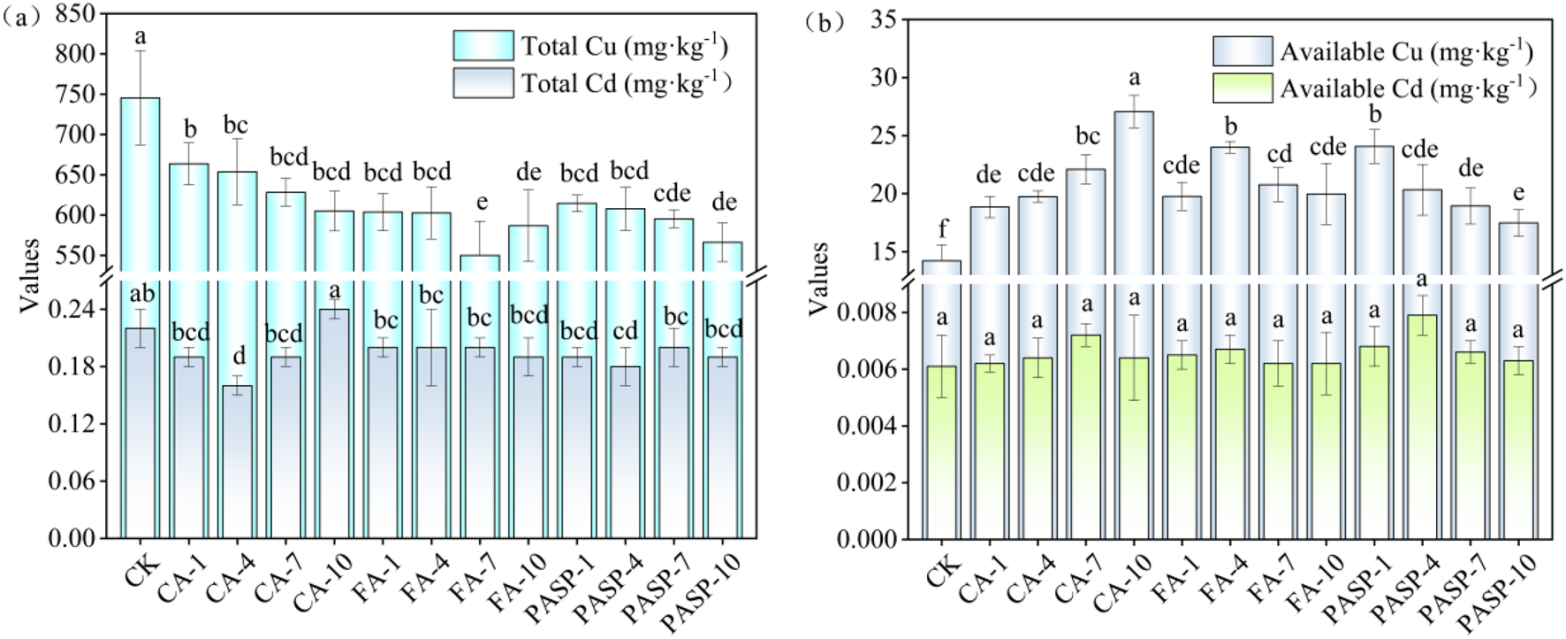
Effects of different treatments on copper tailings total content of heavy metal (**a**), available content of heavy metal (**b**). Values are means ± SD (n = 3). Different letters indicate significant differences at *p* ≤ 0.05 according to the Tukey’s HSD test; values are in the order a > b > c.

As is clear from Fig. 2a, different chelating agents produced varying effects on the total Cu and Cd contents of copper tailings. With the heightening of CA and PASP concentrations, the total Cu contents of copper tailings decreased, all of which differed significantly from the CK group. After the FA incorporation, the total Cu contents of copper tailings were 19.02–26.12% lower than the CK group, with the FA-7 treatment exhibiting the greatest influence over the total Cu content, showing a decrease by 195.45 mg/kg. Meanwhile, the CA-4 and PASP-4 treatments had the most significant effects on lowering the total Cd content of copper tailings, showing decreases by 27.27% and 18.18%, respectively, in comparison to the CK group. Also compared with the CK group, the total Cd content of tailings treated with FA-10 decreased by 13.64%, while no significant changes were noted in the remaining treatment groups. Figure 2b reflects the inter-treatment differences in the available Cu and Cd contents of copper tailings. The incorporation of CA, FA and PASP could all increase the available Cu content of tailings by 32.56–90.30%, 39.03–68.71% and 22.93–69.27%, respectively, compared to the CK group.

### Fraction distribution of heavy metals

**Figure 3:**
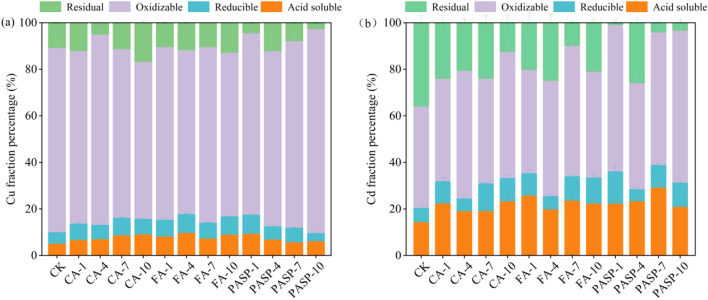
Effects of different treatments on the fraction distribution of Cu (**a**) and Cd (**b**). Values are means ± SD (n = 3). Different letters indicate significant differences at *p* ≤ 0.05 according to the Tukey’s HSD test; values are in the order a > b > c.

It is clear from Fig. 3 that different chelating agents produced varying effects on the morphology of Cu and Cd in copper tailings. The main existing form of Cu in the CK group tailings was oxidizable state (accounting for 79.24% of total Cu), followed by the residual (10.73%), weak acid soluble (5.11%) and reducible (4.92%) states (see Fig. 3a). Under natural conditions, most of the water- and acid-soluble Cd would be converted to more stable forms, such as oxidizable and residual, through the aging effect. As demonstrated by the results of this study, some acid-soluble, oxidizable and reducible Cu and Cd were released into the soil after the chelating agent addition, which facilitated the Cu and Cd absorption by and accumulation in ryegrass compared to the CK group, treatment with CA, FA and PASP increased the content of weak acid soluble Cu by 31.54–78.33%, 43.54–90.29% and 12.38–83.38%, respectively. Except for the PASP-10 treatment, which decreased the content of reducible Cu (accounting for 3.59% of total Cu), the remaining treatments all increased the reducible Cu content to varying degrees. According to the proportion of various forms of Cd in the CK group tailings, the main existing form of Cd in soil was oxidizable state (43.58%), followed by the residual (35.87%), weak acid soluble (14.38%) and reducible (6.17%) states (Fig. 3b). After the chelating agent addition to copper tailings, the general trend was transfer of Cd residual components to more bioavailable components. The percentage of weak acid soluble Cd in tailings treated with various chelating agents increased to varying extents, while the percentage of residual Cd decreased to varying extents. Under PASP-7 treatment, the weak acid soluble Cd content increased the most by 103.37% in comparison to the CK group.

### Heavy metal contents and accumulations in ryegrass

#### Heavy metal contents in ryegrass

**Figure 4:**
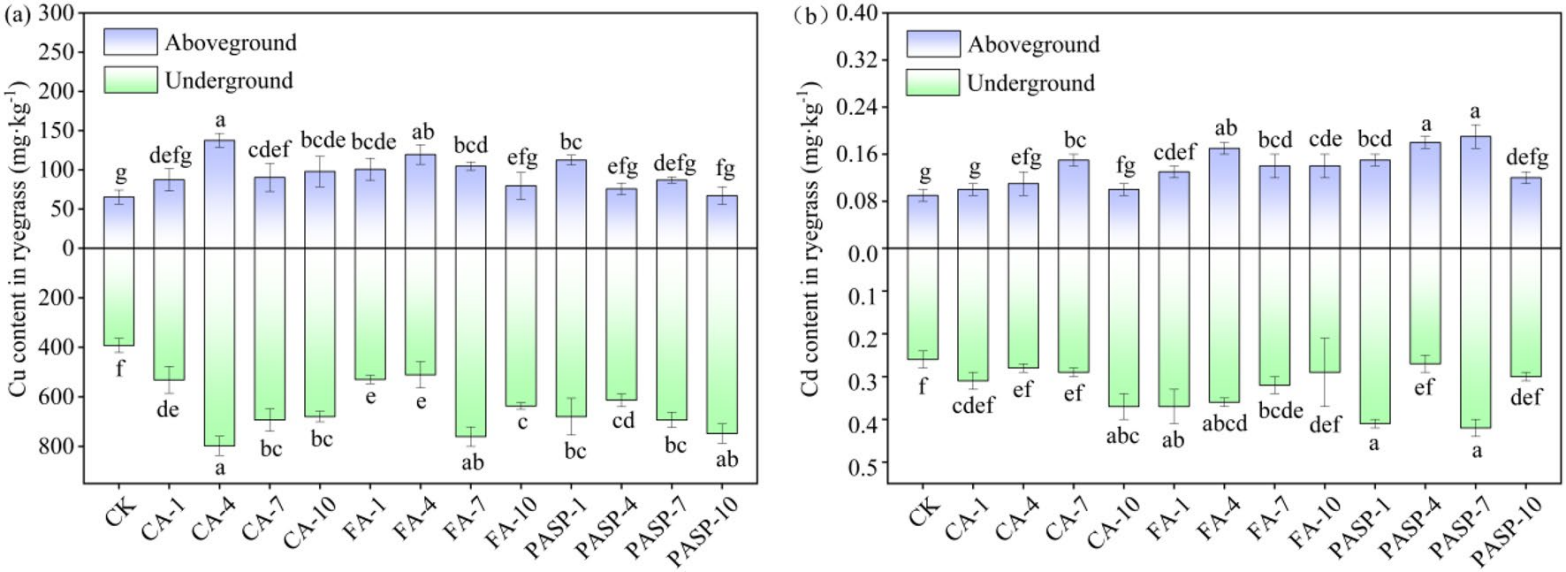
Effects of different treatments on concentration of Cu (**a**) and Cd (**b**) Zn in ryegrass. Values are means ± SD (n = 3). Different letters indicate significant differences at *p* ≤ 0.05 according to the Tukey’s HSD test; values are in the order a > b > c.

With the heightening of CA and FA concentrations, the Cu contents in aboveground and underground parts tended to increase first and then decrease. Under CA-4 treatment, the aboveground and underground Cu contents were all higher than those under other treatments, showing increases by 110.98% and 103.42%, respectively, in comparison to the CK group (see Fig. 4a). With the heightening of PASP concentration, the Cu contents in underground part of ryegrass tended to decrease first and then increase. The Cu contents in aboveground part treated with PASP were 112.45, 75.67, 87.01 and 67.12 mg/kg, respectively. After treatment with CA, FA and PASP, the amounts of Cd absorbed by the aboveground and underground parts all increased, which, compared to the CK group, increased respectively by 11.11–66.67%, 44.44–88.89% and 33.33–111.11% for the aboveground part, whereas by 7.69–42.31%, 18.52–55.56% and 3.85–61.54% for the underground part (see Fig. 4b). Suggestively, the application of CA, FA and PASP to copper tailings could promote the Cd absorption by ryegrass.

#### Accumulation of Cu and Cd in ryegrass

**Figure 5:**
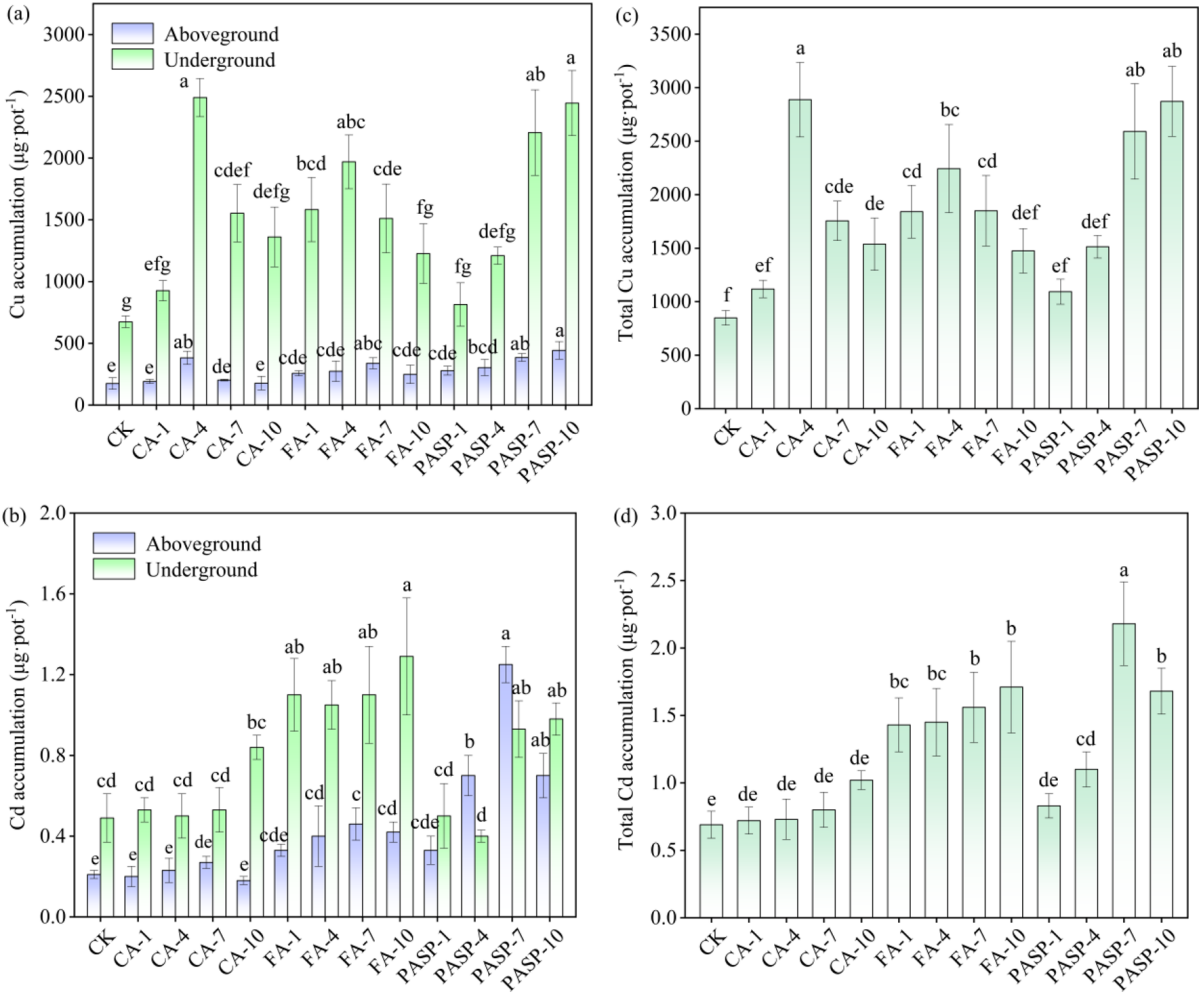
Effects of different treatments on the Cu (**a**) and Cd (**b**) accumulation in underground and aboveground of ryegrass. And effects of different treatments on the Cu (**c**) and Cd (**d**) total accumulation concentrations of ryegrass. Values are means ± SD (n = 3). Different letters indicate significant differences at *p* ≤ 0.05 according to the Tukey’s HSD test; values are in the order a > b > c.

Figure 5 displays the amounts of Cu and Cd accumulated in ryegrass after treatment with different concentrations of chelating agents. The aboveground, underground and total accumulations of Cu all tended to increase first and then decrease with the heightening of CA and FA concentrations, while presented an increasing trend with the heightening of PSAP concentration (Fig. 5a,c). This indicates that the chelating agent-assisted Cu accumulations were primarily decided by the Cu content in plant tissues. Under PASP-7 treatment, the Cu accumulation in the aboveground part reached the maximum, all showing increases by 153.38% compared to the CK group. Both the underground and total Cu accumulations peaked under CA-4 treatment, showing respective increases by 269.37% and 240.22% in comparison to the CK group. The aboveground, underground and total Cd accumulations in the CK group were 0.21, 0.49 and 0.69 μg/pot, respectively. The application of CA produced little effect on the aboveground, underground and total Cd accumulations. Under CA-10 treatment, the underground and total Cd accumulations increased the most, showing respective increases by 71.43% and 47.83% in comparison to the CK group. Under FA treatment, the aboveground, underground and total Cd accumulations were all higher than the CK group, showing respective increases by 57.14–119.05%, 114.29–163.27% and 107.25–147.83%. As the addition of PASP increased, the aboveground and total Cd accumulations exhibited an identical variation trend of initial increases and subsequent decreases, which were the largest under PASP-7 treatment, with values 5.95 and 3.15 times those of the CK group, respectively.

### Remediation efficiency of ryegrass on heavy metals and its potential

**Figure 6:**
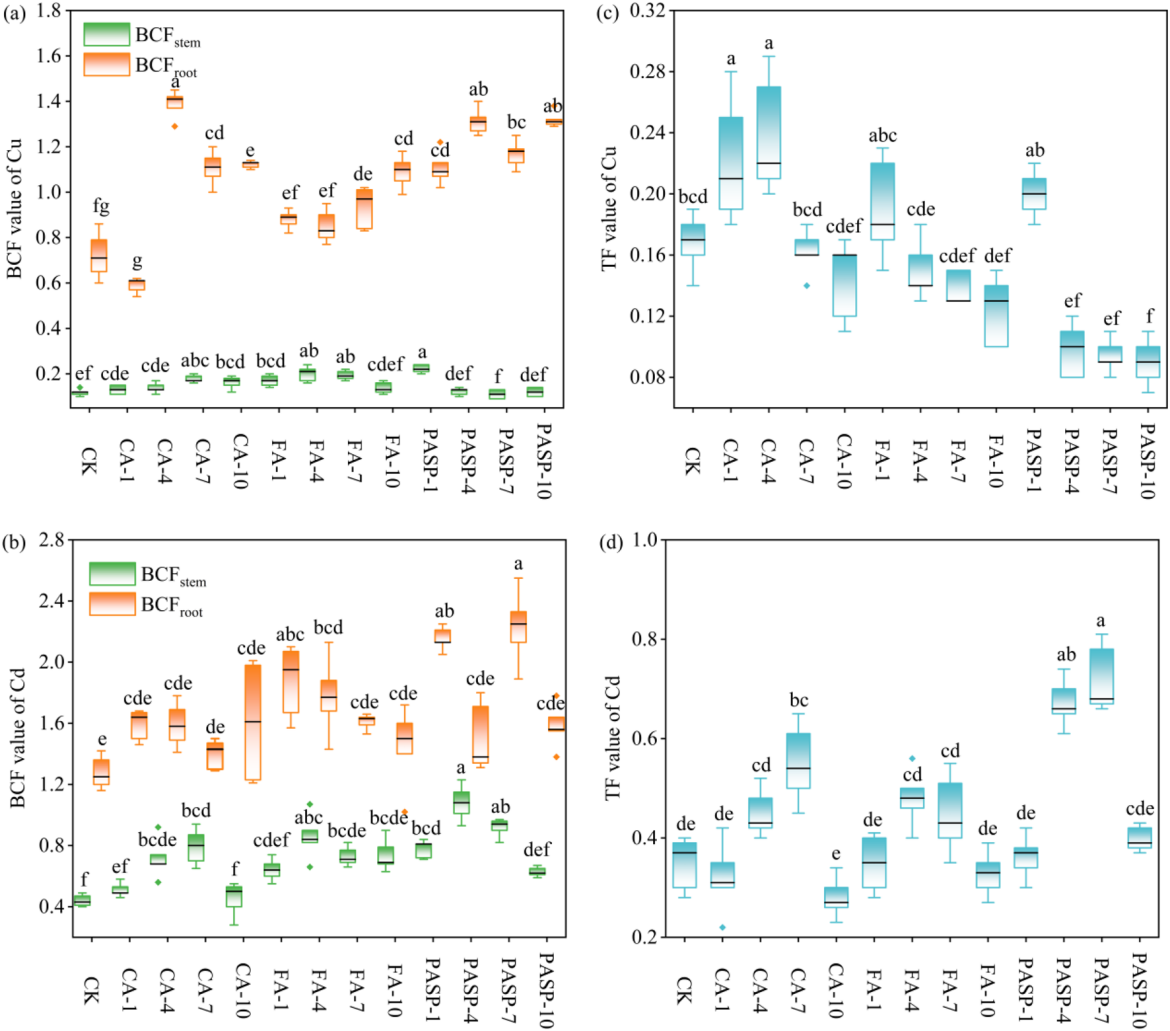
Effects of different treatments on BCF (bio-concentration factor) of Cu (**a**), Cd (**b**) and TF (transfer factor) of Cu (**c**), Cd (**d**). The solid line is the median, the box represents the upper and lower quartiles and whiskers are the 10th and 90th percentiles. Values are means ± SD (n = 3). Different letters indicate significant differences at *p* ≤ 0.05 according to the Tukey’s HSD test; values are in the order a > b > c.

To better evaluate the application effects of CA, FA and PASP for the heavy metal phytoremediation, the heavy metal BCF and TF of various treatment groups were calculated (Fig. 6). With the heightening of CA concentration, the BCF and TF of Cu in ryegrass stems and roots displayed an identical variation trend of initial increases and subsequent decreases, and the BCF and TF values of Cu in roots were the maximum under CA-4 treatment, which were significantly higher by 92.24% and 41.67%, respectively, compared to the CK group. Meanwhile, the BCF of Cu in stems was the minimum under PASP-7 treatment, which was significantly lower than that in the CK group. The remaining treatment groups all differed insignificantly from the CK group, with the PASP-1 treatment exhibiting a relatively large value. The TF value of Cu presented a decreasing trend with the heightening of FA and PASP concentrations, which was the minimum under PASP-10 treatment, showing a significant decrease by 86.67% compared to the CK group. As for the BCF and TF values of Cd in stems, they all tended to increase first and then decrease with the heightening of CA, FA and PASP concentrations. The stem BCF of Cd was the maximum (1.08) under PASP-4 treatment, while the remaining treatment groups all differed insignificantly from the CK group. The root BCF of Cu values of Cd were the largest under PASP-7 treatment, which were 18.00% and 106.90% higher than the CK group, respectively. In summary, different chelating agents produced varying effects on the Cu and Cd absorption in different ryegrass parts.

### Principal component analysis (PCA) and pearson correlation matrix

**Figure 7:**
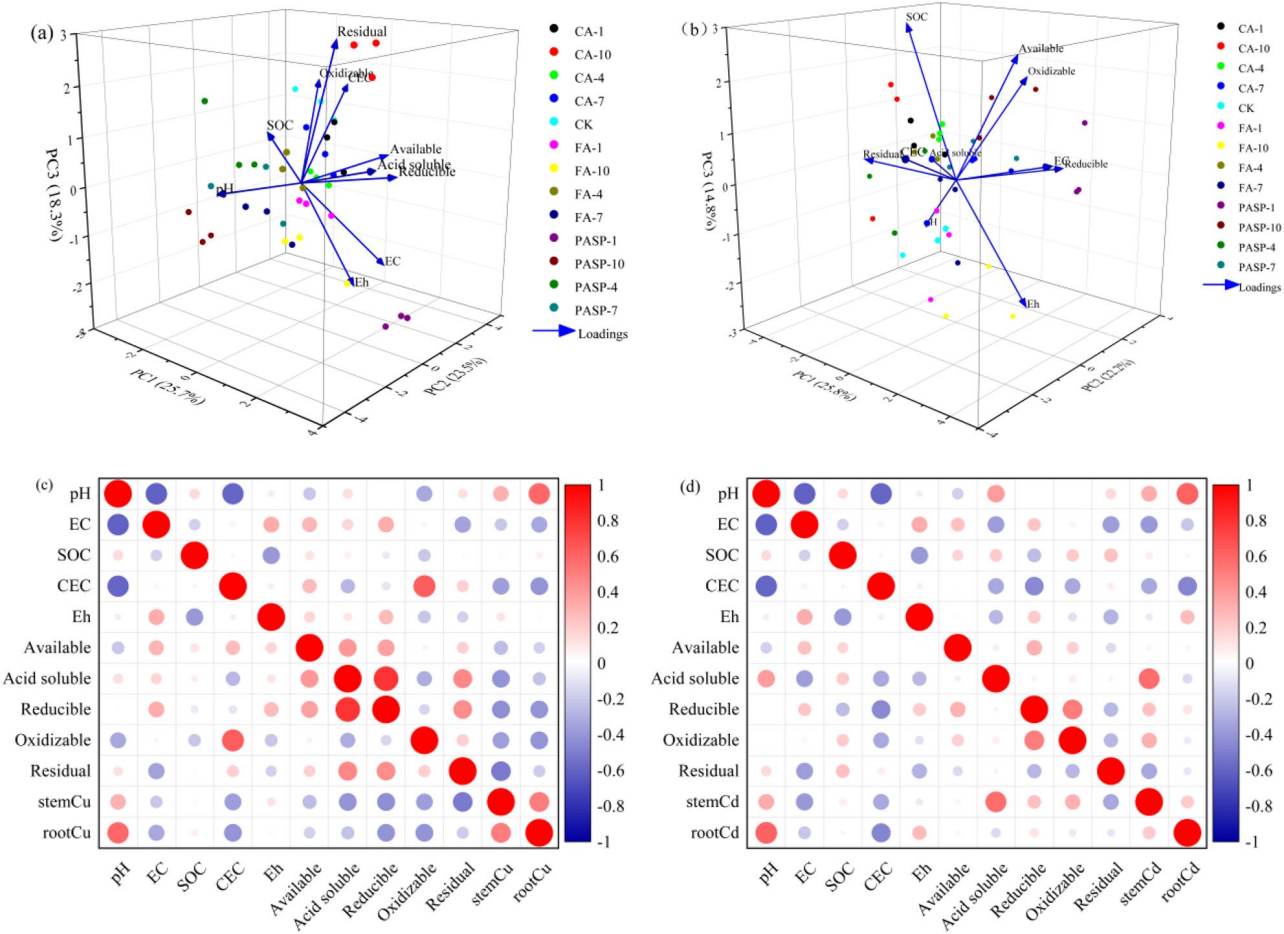
Principal component analysis (PCA) of physiological, biochemical parameters under the application of CA、FA and PASP (**a**). Heat map generated from Pearson’s correlation coefficients of different indices (**b**). EC, electrical conductivity; SOC, soil organic matter; CEC, cation exchange capacity; Eh, redox potential; root Cu, Stem Cu, root Cd, stem Cd represent the concentrations of Cu and Cd in ryegrass root and stem, respectively. Values are means ± SD (n = 3). Different letters indicate significant differences at *p* ≤ 0.05 according to the Tukey’s HSD test; values are in the order a > b > c.

For various treatment groups, the physicochemical properties of copper tailings and the available state and compositionl changes of heavy metals were evaluated by PCA under application of three chelating agents. As is clear from Fig. 7a, the first principal component (PC1), second principal component (PC2) and third principal component (PC3) accounted for 25.7%, 23.5% and 18.3% of the total variance, respectively. According to the PCA results, treatment groups differed significantly from the CK group regarding the distributions of various indices. Additionally, the CEC of copper tailings and available Cu and Cu components (weak acid soluble, reducible, oxidizable and residual) of heavy metals all clustered around different concentrations of CA treatments, and these CA groups all fell on the positive axes of PC1, PC2 and PC3. Suggestively, the treatment with CA was significantly positively correlated with the bioavailability of Cu in copper tailings. On the other hand, the available, weak acid soluble and reducible Cu were more concentrated in the CA-4 group, suggesting that treatment with CA-4 could better improve the Cu bioavailability in copper tailings and promote the phytoabsorption of Cu. It is clear from Fig. 7b that the CK group was located in the lower region of the figure, while the treatment groups other than FA-1 and FA-10 were located in the upper region, showing significant differences from the CK group. The major influencing factors in the PC1 direction included EC, Eh, available Cd, reducible Cd and oxidizable Cd. The PASP-7 treatment fell on the positive axes of PC1, PC2 and PC3, while the EC of copper tailings, available Cd, reducible Cd and oxidizable Cd clustered around the PASP-7 treatment. This indicates that the PASP-7 group had a higher EC value, as well as higher contents of available, reduceable and oxidizable Cd, which were conducive to the phytoextraction of Cd from copper tailings. This is consistent with the earlier conclusion that CA-4 and PASP-7 could more effectively promote the absorption and transport of Cu and Cd, respectively.

Figure 7c,d depict the correlations among the physicochemical properties of copper tailings and the components and accumulations of heavy metals. The Cu contents in roots and stems were negatively correlated with the forms of Cu and CEC. At a large CEC, the negative charge in soil would increase with the increase of CEC, thus providing more adsorption sites to play a role in fixing Cu. The pH of copper tailings was significantly positively correlated with the amount of root accumulation, while was negatively correlated with the available Cu content. Soil pH mainly affected the charge of soil colloids, i.e. affecting the contents of heavy metals by influencing the competition for colloid adsorption sites between heavy metal ions and cations. Soil EC was positively, but not significantly, correlated with the Eh, available Cu, weak acid soluble Cu and reducible Cu. Meanwhile, weak acid soluble Cd was significantly positively correlated with the Cd content in stems, whereas residual Cd was significantly negatively correlated with the Cd concentration in stems. Suggestively, the phytoaccumulation of heavy metals was significantly correlated with the metal components in copper tailings, and the weak acid soluble state was the dominat form of phytoaccumulated metals. A positive correlation was found between the available Cd and the SOM. The pH of copper tailings was positively correlated with the root and stem Cd contents and weak acid soluble Cd, while was significantly negatively correlated with the CEC of copper tailings.

## Discussion

The chelating agent application promoted the plant growth and development to varying degrees, and such an effect varied on different plant parts. He et al.^45^ found that PASP significantly improved the growth of *Solanum nigrum* L. and the accumulation of Cd and Pb. In this study, treatment with PASP-7 and PASP-10 significantly increased the aboveground and underground biomasses of ryegrass, while treatment with CA and FA had unobvious effects. Liu et al.^46^ discovered that PASP, as an amino acid polymer composed of C, H, O and N elements, was well biodegradable and non-toxic to organisms, which was not only applicable as a nutrient enhancer and a metal ion chelating agent, but could also enrich N, P, K and trace elements to supply plants, enabling more effective nutrients utilization by plants to increase their biomass.

Soil pH is a factor that can significantly affect soil nutrient uptake, not only plant growth, but also metal bioavailability, plant uptake of heavy metals, and thus remediation^47^. The pH increase after CA incorporation to copper tailings might be related to the buffering effect of the tailings. Copper tailings often contain large amounts of carbonate, which would react with H^+^ dissociated from CA. Besides, a part of H^+^ would be adsorbed by the anionic sites on the fine tailings particles, leading to a large decrease in H^+^ to somewhat elevate the soil pH^48^. The increase in copper tailings pH after the PASP application might be associated with the nature of the chelating agent itself^49^. As an alkaline chelating agent, the application of PASP would result in the pH elevation of copper tailings. Soil conductivity is related to the salinity in the soil and is an important factor affecting the chemical, physical and biological properties of the soil, as well as an important indicator of soil fertility^50^. A soil conductivity value that is too high or too low values of soil conductivity can hinder plant growth. The increase in EC of copper tailings under chelating agent treatment was probably attributable to the activation of heavy metal ions and other mineral ions in copper tailings by the chelating agents, which heightened the contents of absorbable heavy metal ions and other mineral ions in the tailings to result in the increased tailings EC^51^. Another important factor affecting the uptake of heavy metals in plants is soil organic matter. On the one hand, the soil heavy metal potency is influenced by the soil organic matter content. On the other hand, soils with high organic matter content have a greater supply of nutrients and can better promote plant growth^52,53^. The application of CA, FA and PASP all increased the SOM content of copper tailings to varying degrees. This was probably attributable to the substantial carboxyl and amide groups contained in chelating agent molecular chains. Through their adsorption and chelation functions, the ion absorption could be enhanced, the nutrient loss could be reduced and the soil nutrient accumulation could be promoted, thereby effectively improving the availability of soil nutrients^54^.

Cation exchange capacity can reflect the buffering capacity of the soil, and higher cation exchange contributes to the stabilization of aggregates in the soil^55^. It has been shown that the cation exchange capacity is related to the uptake of heavy metals by plants, and that high cation exchange capacity will block the entry of heavy metals into plants^56^. The addition of chelating agent treatments all increased soil cation exchange to varying degrees. Suggestively, the addition of chelating agents improved the activity of metal ions in copper tailings solution, and enhanced the nutrient preserving and supplying capabilities of the tailings^57^. As for Eh, it is an index reflecting the redox status in soil solution, which is linked to the degree of soil aeration and the availability of soil nutrients^58^. It has been shown that at low redox potentials, Cd precipitates as cadmium sulfide and the amount of Cd absorbed by plants is significantly reduced^59^. The addition of chelating agent to copper tailings can effectively enhance the Eh of copper tailings, but the enhancement decreases with the increase of chelating agent concentration.

Additionally, the available Cu content increased with the heightening of CA concentration, while decreased with the heightening of PASP concentration. The probable reason for the CA concentration-dependent increase in the available Cu content was that the low concentration CA was easily adsorbed by soil particles, since it was mainly composed of single molecules, leading to formation of micelles when its concentration rose beyond the critical concentration of micelles. Such micelles surrounded heavy metals among multiple CA molecules, preventing the metal recombination with soil particles^60,61^. Hence, higher concentration CA had stronger ability to activate soil heavy metals. Addition of the chelating agents also increased the available Cd content in copper tailings. Under CA treatment, the available Cd content of tailings increased by 1.64–18.03% compared to the CK group. The probable reason was that CA could desorb heavy metals in the complexed solid phase particles of soil, subsequently diffusing and transferring them to the liquid phase of soil, thereby enhancing the heavy metal activity^62^. Nevertheless, treatment with FA-7 and FA-10 made the available Cd content of tailings lower than that in the CK group. This might be related to the donor "–COOC–" existing in small molecular organic acids, which was also probably attributable to the easy adsorption of low concentration FA by clay and organic matter^63^. Thus, only within a certain concentration range, a higher concentration was indicative of a better efficiency of FA in activating heavy metals. Compared to other treatment groups, the available Cd content increased the most (0.0079 mg/kg) in the PASP-4 group, possibly because PASP had plentiful carboxyl and hydroxyl groups for heavy metal chelation, which could directly activate the bioavailability of heavy metals in copper tailings^64^.

The above results suggested that the chelating agent addition could increase the weak acid soluble Cu and Cd contents in copper tailings (Fig. 3). The Cd in tailings converted from residual components to more mobile and bioavailable ones, and the effects of CA and PASP treatments were the most prominent. After application, the chelating agents bonded to the dissolved Cu and Cd in soil, causing reduction of free Cu and Cd ions in the soil solution, thereby breaking the precipitation-dissolution equilibrium and promoting the conversion of other forms of Cu and Cd to the weak acid soluble state to establish a new precipitation-dissolution equilibrium^65,66^. Meanwhile, the Cu-chelating agent and Cd-chelating agent complexes were soluble and also in a weak acid soluble state, so the chelating agent application increased the contents of weak acid soluble Cu and Cd in soil^67^. The stronger the complexing power of chelating agent, the more significant the content increase of acid soluble Cu and Cd. Various chelating agents differed in effect and persistence for enhancing the activity of Cu and Cd in soil, which was linked to the chelating agent type. Parveen et al.^68^ found that the CA application to the *Corchorus capsularis* L. seedlings in Cu-contaminated areas was effective in promoting the Cu extraction by the seedlings, as well as their resistance to Cu. CA could exploit unsaturated functional groups to chelate organically bound metals, leading to the increased content of weak acid soluble metals in soil and the decreased content of oxidizable metals^69^. On the other hand, CA could also contribute slightly to the reduction of residual metal content in soil. With the increase of CA, the H^+^ ions in soil solution also increased in concentration, which could not only dissolve insoluble metals to release more metal ions, but also occupy the adsorption sites on the mineral surfaces to replace metal ions, resulting in reduced content of oxidizable metals. Zhang et al.^70^ discovered that FA increased the bioavailability of Cd and Zn in polluted sediments, while reduced the bioavailability of Pb. Through chemical reactions like acidification, complexation, precipitation and redox, FA could directly alter the solubility of heavy metals, allowing conversion of residual metals from stable to active states to improve their bioavailability, thereby promoting the remediation of heavy metal contaminated soil^71^ Kang et al.^72^ found that the application of PASP could significantly improve the Cd extraction capacity and aboveground biomass of *Pennisetum alopecuroides*, with an increase in the weak acid soluble Cd content in soil to 0.092 mg kg^−1^. As a chelating agent, PASP formed a water-soluble metal-chelating agent complex primarily by chelating heavy metal ions in soil solution, thereby altering the occurrence form of heavy metals in soil^73^. Accordingly, the heavy metals were desorbed from the soil particle surfaces to convert from an insoluble to a soluble state, so that they were greatly activated in soil and their bioavailability was improved.

As is clear from Fig. 4, the application of chelating agents could promote the Cu and Cd absorption by various ryegrass parts, and under different treatments, the Cu and Cd contents in various parts manifested a trend of underground part > aboveground part. The underground part absorbed richer Cu and Cd, while the aboveground part absorbed less. This could be explained by the theory of crops' "dilution effect" on heavy metal contents. It holds that for a plant growing in heavy metal contaminated soil, when the weight of its underground part changes little, while the weight of its aboveground part increases evidently, the heavy metal contents therein will be relatively low due to the poor mobility of heavy metals from the plant underground to aboveground parts^74^. This may also be related to the heavy metal absorptive and inhibitory effects of plant itself. The more the heavy metals absorbed by the underground part, the more obvious the transport inhibition in the plant body, so that more absorbed heavy metals are retained in the underground part, with less transportation to the aboveground part^75,76^. The Cu contents in aboveground and underground parts of ryegrass were positively correlated with the CA concentration within a certain range, while an excessively high concentration (7 mmol L^-1^) would decrease the aboveground and underground Cu contents instead (Fig. 4a). Probably, this phenomenon was determined by the combined effects of the heavy metal activation by CA and the absorption of phytoavailable Cu in soil solution by aboveground and underground parts^77^. Compared to the CK group, the Cu and Cd contents in the underground and aboveground parts all increased to certain extents after the chelating agent incorporation into copper tailings. This was primarily because the chelating agents contained ligands like hydroxyl or carboxyl groups that could provide empty orbitals, thus enabling formation of stable soluble chelates with Cu and Cd^78^. Moreover, Cu and Cd entering the soil solution could be effectively absorbed and accumulated by ryegrass, resulting in increased Cu and Cd contents in both the underground and aboveground parts. The chelating agents could increase the Cu and Cd contents in aboveground part, which was probably attributable to the ability of chelating agents to enhance the heavy metal solubility in copper tailings, thereby promoting the heavy metal absorption and accumulation in plant leaves^79^. However, after exceeding certain chelating agent concentrations, the aboveground Cu and Cd levels declined, possibly due to the heavy metal poisoning caused by excessive Cu and Cd chelates in the plant body, subsequently resulting in inhibited accumulation and transport of Cu and Cd^80^.

Unlike other treatments, the ryegrass treated by CA had a significantly weaker ability to extract Cd, which was primarily ascribed to the structure and chemical properties of CA itself. Treatment with a chelating agent containing many carboxyl groups would promote the formation of ligand–metal ion complexes and metal chelates^81^, thus indirectly leading to the dissolution of soil Cd, which was a rather consistent result with that of Kim et al.^82^. As previously reported by Li et al.^83^, PASP has a stronger complexing capacity for Cd than for Cu, which could also explain the varying effects of chelating agents on different metal types. In summary, the incorporation of CA, FA and PASP increased the Cu and Cd accumulations in the aboveground and underground parts of ryegrass. The accumulation of metals in plants was closely associated with the metal content in copper tailings. The above results (see Fig. 3) indicate that treatment with different concentrations of CA, FA and PASP increased Cu and Cd that were utilizable by organisms. Thus, presumably, the increased Cu and Cd accumulations in ryegrass was attributed to the increase of bioavailable Cu and Cd.

The application of chelating agents increased the Cu and Cd accumulations in ryegrass to varying degrees (see Fig. 6a,b), possibly because they increased the concentrations of available heavy metals in soil solution by activating heavy metals in soil^84^. After being captured and absorbed by plant roots, the heavy metals in soil would freely diffuse into the apoplasts of roots, and then enter the plant aboveground part through the root pressure and transpiration effects to be stored in vacuoles, thereby elevating the concentrations of heavy metal ions in the aboveground part to increase the plant BCF^85,86^. Treatment with CA-4 and PASP-7 best promoted the Cu and Cd accumulation in roots, respectively, while PASP-1 and PASP-4 treatments had the respective best effects on promoting the Cu and Cd accumulation in stems. Probably, this was associated with the different nutrients produced by degradation with different chelating agents or the different types of activated nutrients in soil, which led to large disparities in the concentrated Cu and Cd amounts across various parts of ryegrass^87^. Additionally, CA, FA and PASP produced varying transport effects on different heavy metals, suggesting that the chelating agents were selective in promoting the ryegrass phytoextraction of heavy metals from copper tailings in the presence of multiple heavy metals. Except for treatment with CA, other treatments all failed to significantly promote the Cu transport from roots to stems, possibly because CA contained multiple ligands and many active groups as a low molecular organic acid. After addition to soil, it could bond to heavy metals through complexation to form water-soluble complexes, which improved the activity and migration of heavy metals to promote their migration from plant roots to stems. On the other hand, except for PASP which significantly promoted the Cd transport from roots to stems, the remaining chelating agent treatments little impacted the Cd transport effect in copper tailings. The probable reason was that PASP hindered the adsorption and precipitation of Cd on roots by forming a PASP-Cd complex with Cd in soil, which was more easily transported to the stems^88^. Accordingly, the upward Cd transport with transpiration pull was better facilitated, resulting in more accumulation of Cd in the ryegrass stems^89^.

## Conclusions

Currently, the application research of chelating agent-assisted phytoremediation technology in the ecological restoration of mine areas is still in its early stages, especially in terms of the study of chelating agent-assisted phytoremediation technology in soils of copper sulfide mining areas, which has not been reported yet. In summary, the results of this study show that ryegrass has the ability to accumulate heavy metals such as copper and cadmium, which mainly accumulate in its roots. After the application of different types of chelating agents, the remediation efficiency of ryegrass for heavy metal copper and cadmium pollution in copper tailings was enhanced. On the one hand, the application of chelating agents can promote plant growth by affecting the physicochemical properties of copper tailings, thereby increasing biomass and improving heavy metal remediation efficiency. On the other hand, adding citric acid, fulvic acid, and polyaspartic acid increases the biological availability of Cu and Cd in copper tailings, promoting the extraction of Cu and Cd by ryegrass from copper tailings, further improving the remediation efficiency of ryegrass. This study provides important theoretical references for the application of chelating agents in the plant remediation of Cu and Cd contaminated soils in copper sulfide mining areas, and is of great significance for promoting ecological restoration and reconstruction in copper sulfide mining polluted areas. Influence of other metal ions on the chelating behaviour of biodegradable activator-assisted ryegrass in the extraction of Cu and Cd from copper tailings, and the mechanisms of citric acid, fulvic acid, and polyaspartic acid on different heavy metals in copper tailings need further study.

## Data Availability

All data included in this study are available upon request by contact with the corrosponding author.
